# Supplementary material for: Ploidy in Vibrio natriegens: Very Dynamic and Rapidly Changing Copy Numbers of Both Chromosomes
Source: Genes (Basel). 2023 Jul 13;14(7):1437. doi: 10.3390/genes14071437 (PMC10379091; doi:10.3390/genes14071437)
Supplement: Supplementary file 1 [file genes-14-01437-s001.zip › genes-2282744-supplementary.pdf]

name	sequence
Vn Ch1 Std ori FOR	AGGCATTCCCAGGTGACGTATTCTAC
Vn Ch1 Std ori REV	CGGTACTCTAGATTTGCGTTTGCCAC
Vn Ch1 A1A3 ori FOR	AACGCAGGCGTACGTCCAGCTGTTG
Vn Ch1 A3 ori REV	AACCGCGCTCTGCCGCGAAGATAG
Vn Ch1 Std 17% FOR	GTCGGTTCGTACATTCTCAC
Vn Ch1 Std 17% REV	CCAAGCGAGTCCCAATCAAATC
Vn Ch1 A2 17% FOR	GGCAACGATACCAGAAAGAC
Vn Ch1 A2 17% REV	GCCATTCACGCTTGAAACTC
Vn Ch1 Std 33% FOR	GTCAGCGTAGATGTGCTTAG
Vn Ch1 Std 33% REV	GGCCGTTGTAATGGTATCTC
Vn Ch1 A1 33% FOR	CGCACCGAATACGGAACAAGTC
Vn Ch1 A1 33% REV	GCGCACAATGTCTGGTGATG
Vn Ch1 Std ter FOR	GTAGCGCCGGATATTGCTTATCCAAC
Vn Ch1 Std ter REV	CAAGCGTGTTGTGGTTATTCCGGTAC
Vn Ch1 A6 ter FOR	CCGGACAACGATACGTTGG
Vn Ch1 A6 ter REV	GGGCTTCGTAGTCTTTAGGC
Vn Ch2 Std ori FOR	AGATACCGTTCTTGCTGCTGTTCTAC
Vn Ch2 Std ori REV	TGGTCATCGGAATGTTGACCACAAAG
Vn Ch2 Ana ori FOR	GTCATTGCTCGCCTCAAAGTCTTTAC
Vn Ch2 Ana ori REV	TCGATACAATAGCCATCCATTGCCTG
Vn Ch2 Std 17% FOR	GGTACACGTGCCATCTAGACAG
Vn Ch2 Std 17% REV	ATAGCCCGCCTTCGTTCATC
Vn Ch2 A1 17% FOR	ACTGCCTAGTGTTTCGGATG
Vn Ch2 A1 17% REV	GACAGGGAAGCCACAAACTC
Vn Ch2 Std 33% FOR	ATGTGGCGTTCTCTTTCCTCTC
Vn Ch2 Std 33% REV	TCCTGTGCGTGGGTATTTATCC
Vn Ch2 A1 33% FOR	CGTTGGGTCGGGTAATTTGTAG
Vn Ch2 A1 33% REV	GCAACAGAGGACGTGCTTTATC
Vn Ch2 Std ter FOR	CGATTTGCGGAAGATAACGGTTTCTG
Vn Ch2 Std ter REV	CGATGTCTGATTTTGGACGTCGAATG
Vn Ch1 A6 ter FOR	TAACTTGCTTTTAGCGGCTTTCGGTG
Vn Ch1 A6 ter REV	CCTAATATCCGCAACTTTGCAGACTC

application	annealing temperature in PCR [°C]
amplification of the standard fragment of Ch1 ori	57
amplification of the standard fragment of Ch1 ori	57
amplification of the analysis fragment of Ch1 ori	67
amplification of the analysis fragment of Ch1 ori	67
amplification of the standard fragment of Ch1 17%	51
amplification of the standard fragment of Ch1 17%	51
amplification of the analysis fragment of Ch1 17%	52
amplification of the analysis fragment of Ch1 17%	52
amplification of the standard fragment of Ch1 33%	51
amplification of the standard fragment of Ch1 33%	51
amplification of the analysis fragment of Ch1 33%	55
amplification of the analysis fragment of Ch1 33%	55
amplification of the standard fragment of Ch1 ter	57
amplification of the standard fragment of Ch1 ter	57
amplification of the analysis fragment of Ch1 ter	57
amplification of the analysis fragment of Ch1 ter	57
amplification of the standard fragment of Ch2 ori	56
amplification of the standard fragment of Ch2 ori	56
amplification of the analysis fragment of Ch2 ori	56
amplification of the analysis fragment of Ch2 ori	56
amplification of the standard fragment of Ch2 17%	55
amplification of the standard fragment of Ch2 17%	55
amplification of the analysis fragment of Ch2 17%	52
amplification of the analysis fragment of Ch2 17%	52
amplification of the standard fragment of Ch2 33%	55
amplification of the standard fragment of Ch2 33%	55
amplification of the analysis fragment of Ch2 33%	53
amplification of the analysis fragment of Ch2 33%	53
amplification of the standard fragment of Ch2 ter	56
amplification of the standard fragment of Ch2 ter	56
amplification of the analysis fragment of Ch2 33%	57
amplification of the analysis fragment of Ch2 33%	57
